# Comparison of the treatment efficacy of umbilical mesenchymal stem cell transplantation via renal subcapsular and parenchymal routes in AKI-CKD mice

**DOI:** 10.1186/s13287-022-02805-3

**Published:** 2022-03-25

**Authors:** Mengjie Huang, Duo Li, Jianwen Chen, Yuwei Ji, Tingyu Su, Yulan Chen, Yingjie Zhang, Yuanda Wang, Fei Li, Shang Chen, Yu Dong, Qinggang Li, Lingling Wu, Zhe Feng, Jie Wu, Li Zhang, Zongjin Li, Guangyan Cai, Xiangmei Chen

**Affiliations:** 1 Department of Nephrology, First Medical Center of Chinese PLA General Hospital, Nephrology Institute of the Chinese People’s Liberation Army, State Key Laboratory of Kidney Diseases, National Clinical Research Center for Kidney Diseases, Beijing Key Laboratory of Kidney Disease Research, No.28 Fuxing Road, Beijing, 100853 China; 2 Institute of Disaster and Emergency Medicine, Tianjin University, Tianjin, 300072 China; 3 Wenzhou Safety (Emergency) Institute, Tianjin University, Wenzhou, 325000 China; 4 School of Medicine, Nankai University, Weijin 20 Road, Tianjin, 300071 China; 5 The Key Laboratory of Bioactive Materials, Ministry of Education, Nankai University, The College of Life Sciences, Tianjin, China

**Keywords:** Mesenchymal stem cells, Acute kidney injury, Subcapsular transplantation, Parenchymal transplantation, Collagen I

## Abstract

**Background:**

Mesenchymal stem cells (MSCs) have emerged as a promising cell-based therapy for acute kidney injury (AKI). However, the optimal route of MSC transplantation remains controversial, and there have been no comparisons of the therapeutic benefits of MSC administration through different delivery routes.

**Methods:**

In this study, we encapsulated MSCs into a collagen matrix to help achieve local MSC retention in the kidney and assessed the survival of MSCs in vitro and in vivo. After transplanting collagen matrix-encapsulated-MSCs (Col-MSCs) under the renal capsule or into the parenchyma using the same cell dose and suspension volume in an ischemia/reperfusion injury model, we evaluated the treatment efficacy of two local transplantation routes at different stages of AKI.

**Results:**

We found that Col-MSCs could be retained in the kidney for at least 14 days. Both local MSC therapies could reduce tubular injury, promote the proliferation of renal tubular epithelial cells on Day 3 and alleviate renal fibrosis on Day 14 and 28. MSC transplantation via the subcapsular route exerts better therapeutic effects for renal functional and structural recovery after AKI than MSC administration via the parenchymal route.

**Conclusions:**

Subcapsular MSC transplantation may be an ideal route of MSC delivery for AKI treatment, and collagen I can provide a superior microenvironment for cell–cell and cell–matrix interactions to stabilize the retention rate of MSCs in the kidney.

**Supplementary Information:**

The online version contains supplementary material available at 10.1186/s13287-022-02805-3.

## Introduction

Acute kidney injury (AKI) is a common and severe clinical syndrome characterized by a rapid decline in renal function due to ischemia/reperfusion injury (IRI), nephrotoxins, sepsis and other causes [1]. As the kidney has a limited repair capacity, persistent or severe injury can result in the failure of renal repair and the transition to chronic kidney disease (CKD) [2]. According to a meta-analysis of 13 cohort studies, AKI increases the risk of CKD by 8.8-fold and the risk of end-stage renal disease (ESRD) requiring replacement therapies by 3.3-fold [3]. These data point toward an urgent need to identify therapeutic interventions to treat AKI and prevent AKI from progressing to CKD.

In recent years, mesenchymal stem cells (MSCs) have emerged as a promising novel therapeutic method to prevent or ameliorate AKI [4–7]. Although MSCs have been demonstrated to engraft within the kidney and differentiate into renal parenchymal cells, this transdifferentiation occurs at a very low frequency and has limited beneficial effects [8]. The therapeutic efficacy of MSCs is relatively more likely to be mediated by paracrine mechanisms [9]. By secreting cytokines and growth factors, MSCs promote tubular cell proliferation and improve the microenvironment associated with kidney repair [10].

Currently, systemic delivery, such as the intra-arterial (IA) and intravenous (IV) routes, is commonly used for MSC transplantation in AKI treatment. However, the low renal implantation rate, low cell retention rate and high embolism risk limit IV/IA therapies [11, 12].

Local transplantation around the kidney, such as the renal parenchymal and subcapsular routes, can restrict MSCs to the injured region and enhance their paracrine effects, and these methods have attracted widespread attention [13–16]. In addition, local transplantation has few side effects on the body, as the effect remains localized at the site [17]. However, the renal parenchymal and subcapsular routes also have drawbacks. The procedures are more traumatic than other routes of administration, and the surgery is more difficult. To date, few studies have compared the effects of the two local transplantation routes, and the ideal route of stem cell transplantation for AKI treatment remains unknown.

Due to the limited space in the renal parenchyma or under the renal capsule and high internal pressure, MSCs administrated in a liquid state are easily leaked out, and few MSCs can remain in the kidney. Therefore, biomaterials with good biocompatibility are often required to encapsulate MSCs to help achieve local MSC retention in the kidney in a solid form. Collagen I is one of the most commonly used natural biological materials in tissue engineering research. Collagen I has many biological activities in addition to tissue support and is also an important part of the extracellular matrix (ECM) [18–20]. Many studies have reported that collagen matrix is suitable for tissue support to enhance cell attachment and proliferation [21–23].

In this study, we encapsulated MSCs in a collagen matrix to avoid the loss of cells from the transplanted region and explored the in vivo survival of MSCs after administration. We further compared the treatment efficacy of MSC transplantation via the renal subcapsular and parenchymal routes and examined the ideal route of MSC delivery for AKI-CKD treatment.

## Materials and methods

### Cell culture and collagen matrix preparation

Human umbilical cord-derived MSCs (HUC-MSCs) were obtained from Vcanbio Cell & Gene Engineering Corp., Ltd. (Tianjin, China) and were cultured in MSC-conditioned medium (YOCON Biology, Beijing, China). RFP-labeled MSCs (RFP-MSCs) were purchased from Cyagen Biosciences (Cyagen Biosciences, Sunnyvale, CA, USA). MSCs at passages 4 to 6 were used for subsequent experiments. All cells were cultured at 37 °C in a humidified incubator with 5% carbon dioxide (CO_2_) and 95% air.

For collagen matrix preparation, rat tail collagen I (3 mg/ml; Life Technologies, USA) and sterile 10 × phosphate-buffered saline (PBS) were mixed in a 9:1 ratio, and an appropriate volume of 1 M NaOH was added to adjust the pH to 7.4. All operations were performed on ice.

To obtain collagen matrix-encapsulated MSCs (Col-MSCs), 2 × 10^6^ adherent MSCs were digested with trypsin, washed with PBS and resuspended in 80 μl of diluted collagen I matrix solution.

### Animal models and cell-based therapy

Wild-type C57BL/6 mice (8 weeks old) were raised in a specific pathogen-free (SPF) facility at the Animal Center of Chinese PLA General Hospital. For the unilateral ischemia reperfusion injury (uIRI) model, the unilateral renal pedicles (left) in the mice were clipped for 30 min with microaneurysm clamps. During the ischemic period, the core body temperature was monitored and maintained at 37 °C by using a temperature-controlled heating system. Delayed contralateral nephrectomy (right kidney) was performed 1 day prior to sample harvest [24–26]. This uIRI model could induce kidney injury of notable severity without a high mortality rate, permitting assessment of the AKI-to-CKD progression.

After 5 min of reperfusion, 2 × 10^6^ MSCs (per kidney) [16, 27] suspended in collagen matrix were injected under the renal capsule (subcapsular transplantation) or were intrarenally injected into two sites in the left renal cortex (parenchymal transplantation) in a total volume of 80 μL. Supplementary Figure 1A in Additional file 1 shows the schematic of Col-MSC injection under the renal capsule and into the parenchyma. When the subrenal capsule injection is performed, the needle is moved along the tangential direction of the kidney surface with the needle tip facing upwards. A small amount of Col-MSC suspension is injected at the moment that the needle is inserted to create a bulge in the subcapsular cavity, and then the needle is lifted upward to continue the injection. After the injection, the suspension solidified at room temperature, and then the needle was pulled out to avoid leakage.

To further confirm the successfully local delivery of MSCs in the kidney, HE staining and immunohistochemistry analysis of CD73, a HUC-MSC marker, were performed using kidney specimens on Day 1 and 3 post-AKI. CD73-positive MSCs could be observed around the kidney capsule (Additional file 1: Supplementary Figure 1B).

Equal injections of collagen were used as controls. Sham-operated animals were subjected to a similar surgical procedure without IRI or injection.

All animals were handled in strict and the procedures were reviewed and approved by the Institutional Animal Care and Use Committee of the Chinese PLA General Hospital.

### In vivo and in vitro fluorescence imaging analysis

In vivo fluorescence imaging was performed by an IVIS Lumina imaging system (Caliper Life Sciences, Waltham, MA, USA) to investigate the fate of MSCs after transplantation.

After subcapsular or parenchymal RFP-MSC transplantation, the mice were imaged in vivo at the indicated time points (Day 1, 3, 5, 7, 14 and 28) to track the survival of RFP-MSCs in the IRI model. The fluorescence signal of RFP-MSCs is presented as the average radiance (photos/sec/cm2/steradian, p/sec/cm^2^/sr) of the region of interest (ROI) over the kidney after IRI injury [28].

To assess the relationship between the number of MSCs and the fluorescent signals, RFP-MSCs were seeded at different densities in 96-well plates with 100 μL of complete medium and imaged by an IVIS Lumina imaging system. The fluorescence intensity of the cells was assessed in the ROI and quantified as photo flux in units of p/sec/cm^2^/sr with the IVIS® imaging system [29].

### High-content imaging

Col-MSCs were incubated in ultrathin 96-well plates (Perkin Elmer CellCarrier Ultra, 6055302) in high-content imaging system equipment (Operetta CLS, Perkin Elmer) for 84 h at 37 °C. Photos were taken every 20 min to continuously and dynamically observe changes in MSC morphology and proliferation.

### Three-dimensional laser scanning confocal analysis

Three-dimensional laser scanning was performed by a two-photon microscope (TSP-SP5, Leica, Germany) to analyze the survival of RFP-MSCs encapsulated by collagen matrix in vitro. Two-photon fluorescence emission was collected at wavelengths from 555 to 624 nm upon excitation at 543 nm for red (RFP-MSCs). Image acquisition was performed in Z scan automatic volume mode, and a series of 60 images to a depth of 300 μm were captured.

### Histopathological examination and immunohistochemical staining

Kidney tissue was fixed in 4% formaldehyde, dehydrated, and embedded in paraffin. Tissue sections (2 μm) were stained with hematoxylin–eosin (HE), periodic acid-Schiff (PAS) and Masson’s trichrome. Tubular injury was assessed on PAS-stained sections in a blinded manner. The assessment was based on histopathologic changes (i.e., tubular necrosis, cast formation, tubular dilation, and loss of brush borders) that were mainly located at the cortical medullary junction area. Ten nonoverlapping fields (200×) were randomly selected and scored from 0 to 4 (0: normal; 1: mild to moderate injury, involvement of 1–25%; 2: severe injury, involvement of 26–49%; 3: high severe injury, involvement of 50–75%; and 4: extensive injury, involvement of > 75%).

For Masson-stained tissue, the area of each field of view and the area of collagen fibers stained in green were measured using ImageJ software. The relative area of collagen deposition was calculated as follows: area of collagen fibers stained green/area of field of view × 100%.

Immunohistochemical staining was performed as described previously [30].

### Immunofluorescence staining

The kidneys were fixed for 24 h in 4% paraformaldehyde at 4 °C, incubated for 2 h in a 30% sucrose solution and embedded in optimal cutting temperature compound. Each embedded kidney was cut into 5-µm-thick sections and permeabilized with 0.4% Triton X-100 buffer. After being washed with PBS, the sections were blocked with 5% bovine serum albumin and incubated with primary antibodies overnight. The primary antibodies used were as follows: KIM-1 (AF1817, R&D), PCNA (ab92552, Abcam), α-SMA (ab7817, Abcam), fibronectin (ab2413, Abcam), and CD73 (AF 7638, Beyotime Biotechnology). To visualize the primary antibodies, the slides were stained with cyanine FITC- or Cy3-conjugated secondary antibodies (Beyotime Biotechnology, China) for 2 h at room temperature. Fluorescein labeled Lotus Tetragonolobus Lectin (LTL; marker of proximal tubule) were obtained from Vector labs (FLe1321, Vector Laboratories). Finally, 4′,6-diamidino-2-phenylindole (DAPI) was added (ab104139, Abcam). The stained slides were viewed under a Leica TCS-SL confocal microscope.

### Statistical analysis

All data are expressed as the mean ± standard deviation (SD). Statistical analysis was performed using GraphPad Prism software. Comparisons between groups were made using one-way analysis of variance (ANOVA), followed by Student’s t-test. A value of *p* < 0.05 was considered significant.

## Results

### Characterization of Col-MSCs in vitro

**Fig. 1:**
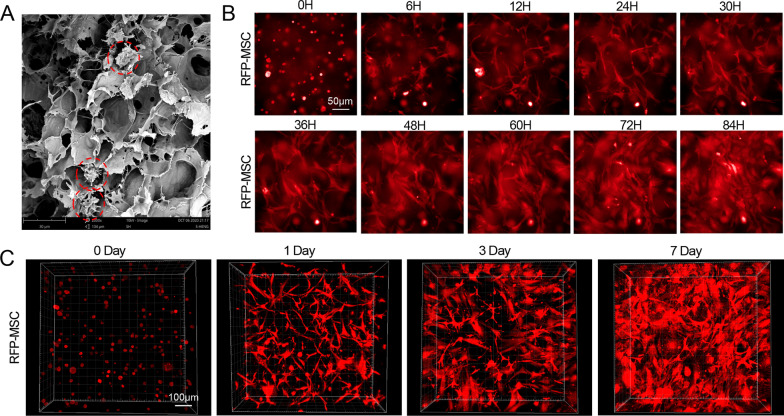
Characteristics of Col-MSCs. **A** Scanning electron microscopy image of Col-MSCs under × 2000 magnification. The red dotted line shows the captured MSCs in the collagen matrix. **B** The change in MSC morphology from 0 to 84 h after the cells were mixed with collagen matrix. Scale bar = 50 μm. **C** Three-dimensional scanning of RFP-MSCs in collagen matrix on Day 0, 3, and 7. Scale bar = 100 μm

Scanning electron microscopy shows that the collagen matrix was a reticulated porous structure that was suitable for cell attachment. MSCs were captured by the crosslinking network (Fig. 1A). The growth and proliferation of RFP-MSCs were observed continuously and dynamically for 84 h by high-content screening after the cells were mixed with collagen matrix. It took nearly 24 h for MSC morphology to change from a round shape just after digestion to a normal spindle shape, and the cells proliferated in the collagen matrix (Fig. 1B and Additional file 2: Supplementary Video 1). To identify the survival time of MSCs in collagen matrix in vitro, RFP-MSCs were also cultured in collagen matrix and scanned in three dimensions at the indicated time points. As shown in Fig. 1C, RFP-MSCs were spherical on Day 0, and the cells were long and spindle-shaped and grew well on Day 3. There were still living cells in vitro until Day 7.

### In vivo retention of Col-MSCs

**Fig. 2:**
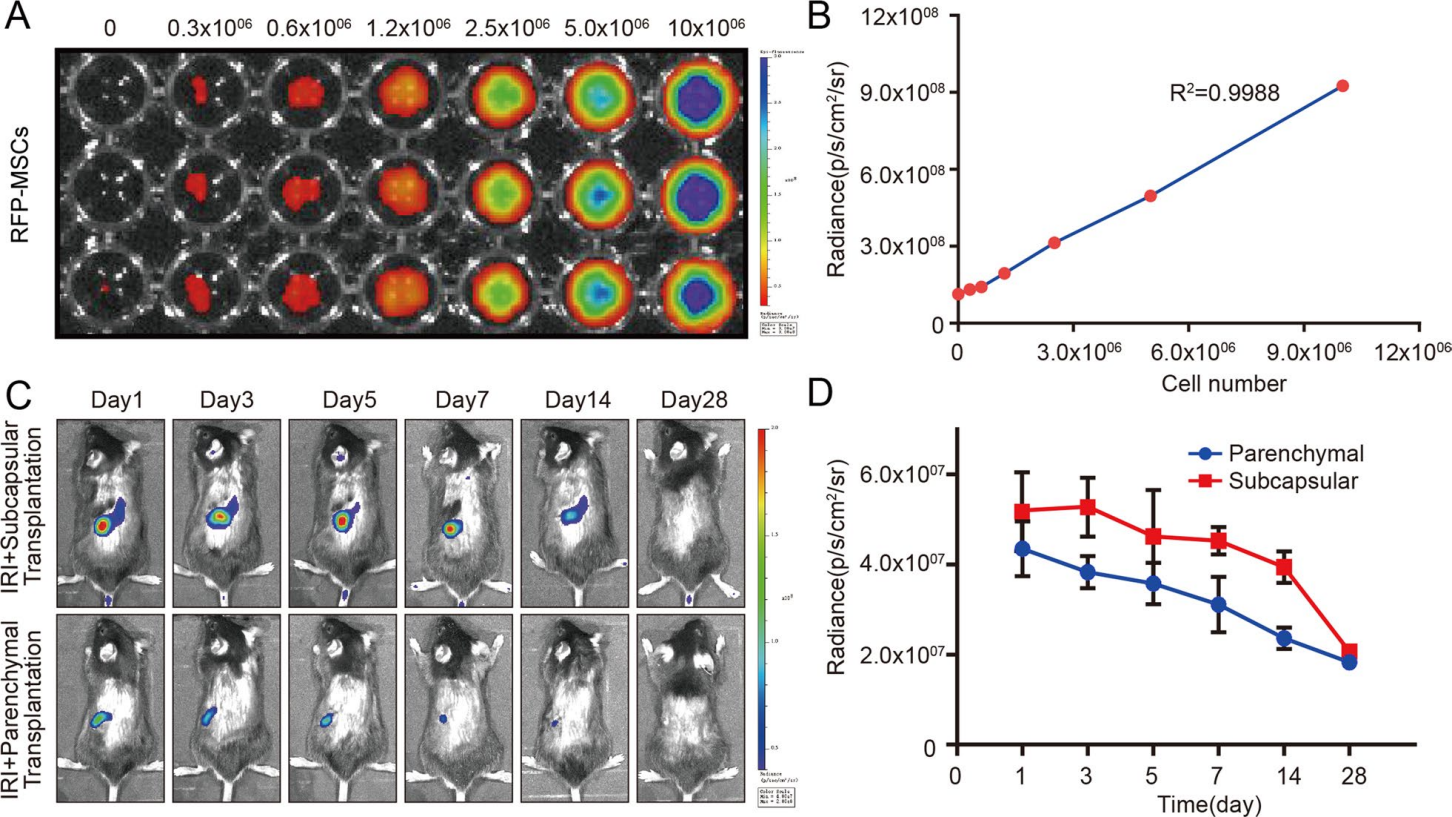
Retention of Col-MSCs after transplantation in vivo. **A**, **B** Correlation of RFP-MSC numbers and fluorescent signals. The experiment was performed in triplicate. **C** Longitudinal tracking of labeled cells over a 28-day period in an IRI mouse model via bioluminescence live imaging. **D** Quantification of the time-dependent fluorescent signal dynamics, as an indicator of cell retention

We used RFP-MSCs to track the fate of transplanted cells in vivo. Fluorescent imaging quantification by a live animal imaging system demonstrated a robust linear correlation between the number of MSCs and signals (*R*^2^ = 0.9988) (Fig. 2A, B), which indicates that fluorescence intensity can be analyzed to assess the retention of MSCs in vivo. After Col-MSCs were transplanted under the renal capsule or into the parenchyma of IRI mice, robust signals were measured on the first day in both groups, suggesting successful transplantation. The fluorescence intensity increased on Day 3 and gradually decreased throughout the experimental period. However, there were still signals 14 days after transplantation (Fig. 2C, D). These results indicate that Col-MSCs can be retained in vivo for a long time. In addition, we found that the overall signal of MSCs under the capsule was stronger than that in the renal cortex (Fig. 2C, D).

### Subcapsular MSC transplantation is superior to parenchymal route in kidney recovery on Day 3

**Fig. 3:**
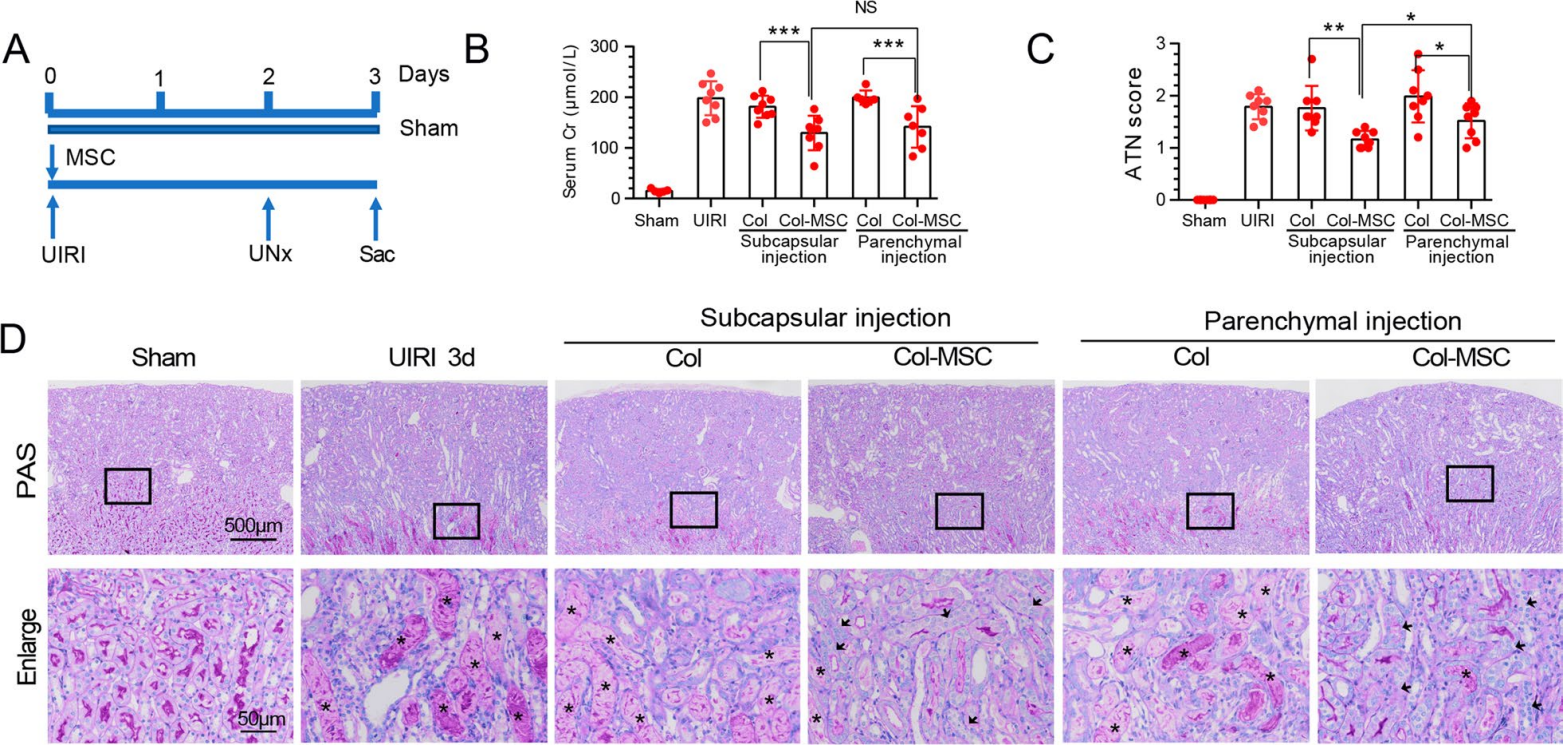
Assessment of renal function and kidney pathological damage after MSC transplantation via the subcapsular and parenchymal routes. **A** Experimental design. **B** Serum creatinine levels in the groups at 3 days after IRI. ***p < 0.001, ANOVA corrected with the Bonferroni coefficient. *n* = 5–8 mice per group. **C** Quantitative assessment of tubular damage. **p < 0.01; *p < 0.05, ANOVA corrected with the Bonferroni coefficient. *n* = 10 fields per mice. **D** Representative micrographs of PAS staining showing kidney injury in IRI mice after subcapsular and parenchymal MSC transplantation. Asterisks in the enlarged boxed areas indicate injured tubules. Arrows in the enlarged boxed areas indicate regenerative cells

**Fig. 4:**
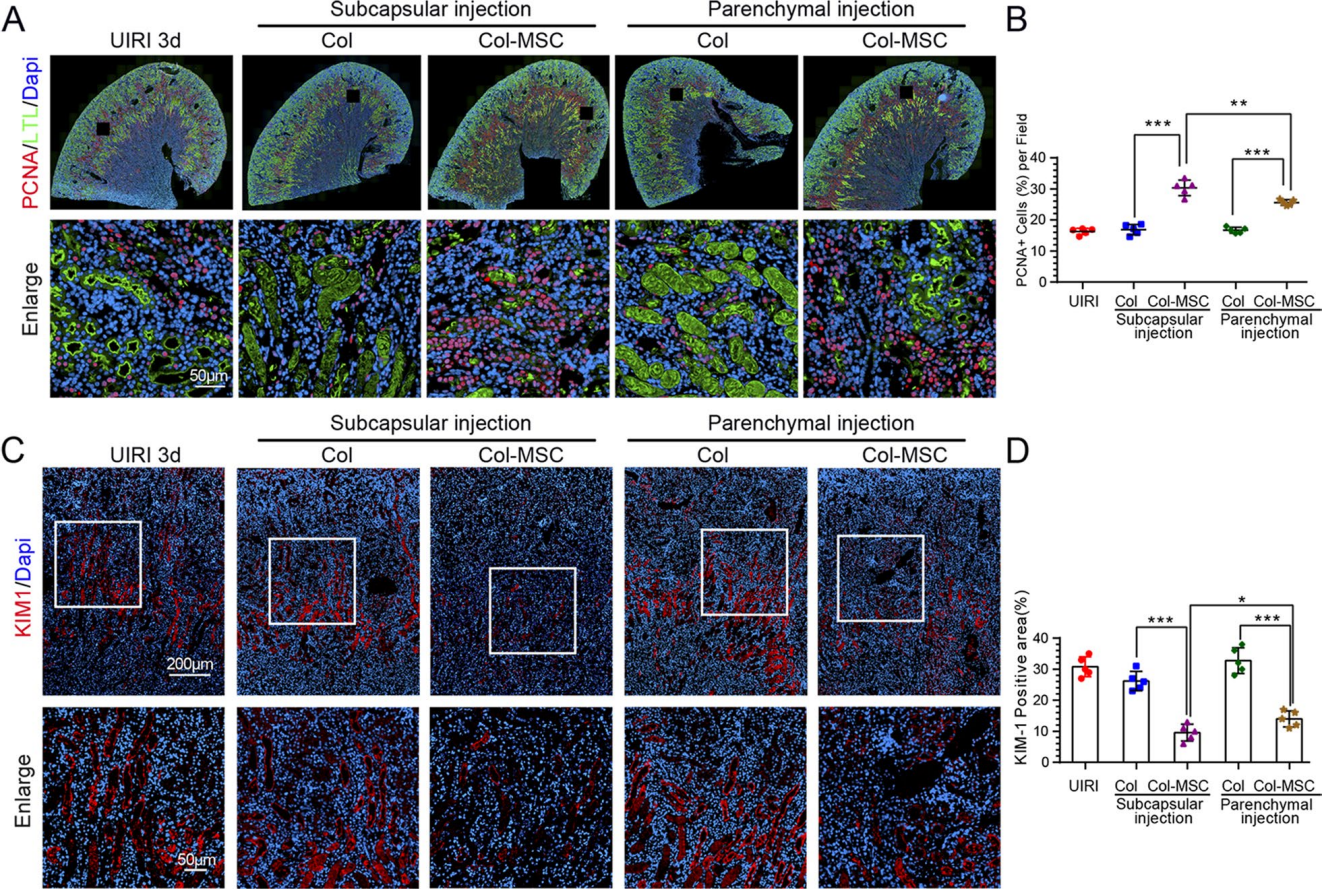
Changes in tubular injury and repair after MSC transplantation via the subcapsular and parenchymal routes. **A** Representative immunofluorescence staining of PCNA (red) and LTL (marker of proximal tubule; green) in the groups after IRI. **B** Quantitative analysis of PCNA-positive cells in the LTL^+^ proximal tubules in the groups after IRI. ***p < 0.001, ANOVA corrected with the Bonferroni coefficient. *n* = 5 per group. **C** Representative immunofluorescence staining of KIM-1 (red) in the groups after IRI. **D** Quantitative analysis showing the KIM-1-positive areas in the groups after IRI. ***p < 0.001, ANOVA corrected with the Bonferroni coefficient. *n* = 5 per group

To investigate the changes in tubular injury and repair after subcapsular and parenchymal MSC transplantation in AKI, IRI mice were sacrificed on Day 3 (Fig. 3A). As shown in Fig. 3B, serum creatinine (SCr) levels were increased significantly in IRI mice compared with sham mice. Notably, SCr levels were decreased in both local Col-MSC transplantation groups compared with the Col groups. However, there was no significant difference in SCr levels between the subcapsular and parenchymal groups (Fig. 3B). PAS staining showed tubular injury, which was characterized by loss of the brush border, tubular cell loss, tubular dilatation and cast formation, which were apparent in the IRI and Col groups, and less morphological injury and significantly increased tubular epithelial cell regeneration and rearrangement were observed in both MSC-transplanted groups. To compare the therapeutic effect of MSCs administered by the two different routes, we further analyzed ATN scores and found that the scores were higher in the parenchymal group than those in the subcapsular group (Fig. 3C, D). We further verified renal tubular cell proliferation by measuring PCNA-positive cells in the LTL^ +^ proximal tubules. The numbers of PCNA-positive cells were significantly increased in the MSC groups compared to the injured and nontransplanted groups. Compared with parenchymal MSC transplantation, subcapsular transplantation induced more PCNA-positive tubular cells (Fig. 4A, B). Moreover, immunofluorescence staining of KIM-1, a biomarker of tubular injury, was performed and showed reductions in the KIM-1-positive areas in the MSC groups at 3 days after IRI, and the percentages of the KIM-1 areas in the subcapsular MSC transplantation groups were lower than those in the parenchymal MSC transplantation groups (Fig. 4C, D).

### Subcapsular MSC transplantation exerts superior antifibrotic effects in AKI-CKD mice

**Fig. 5:**
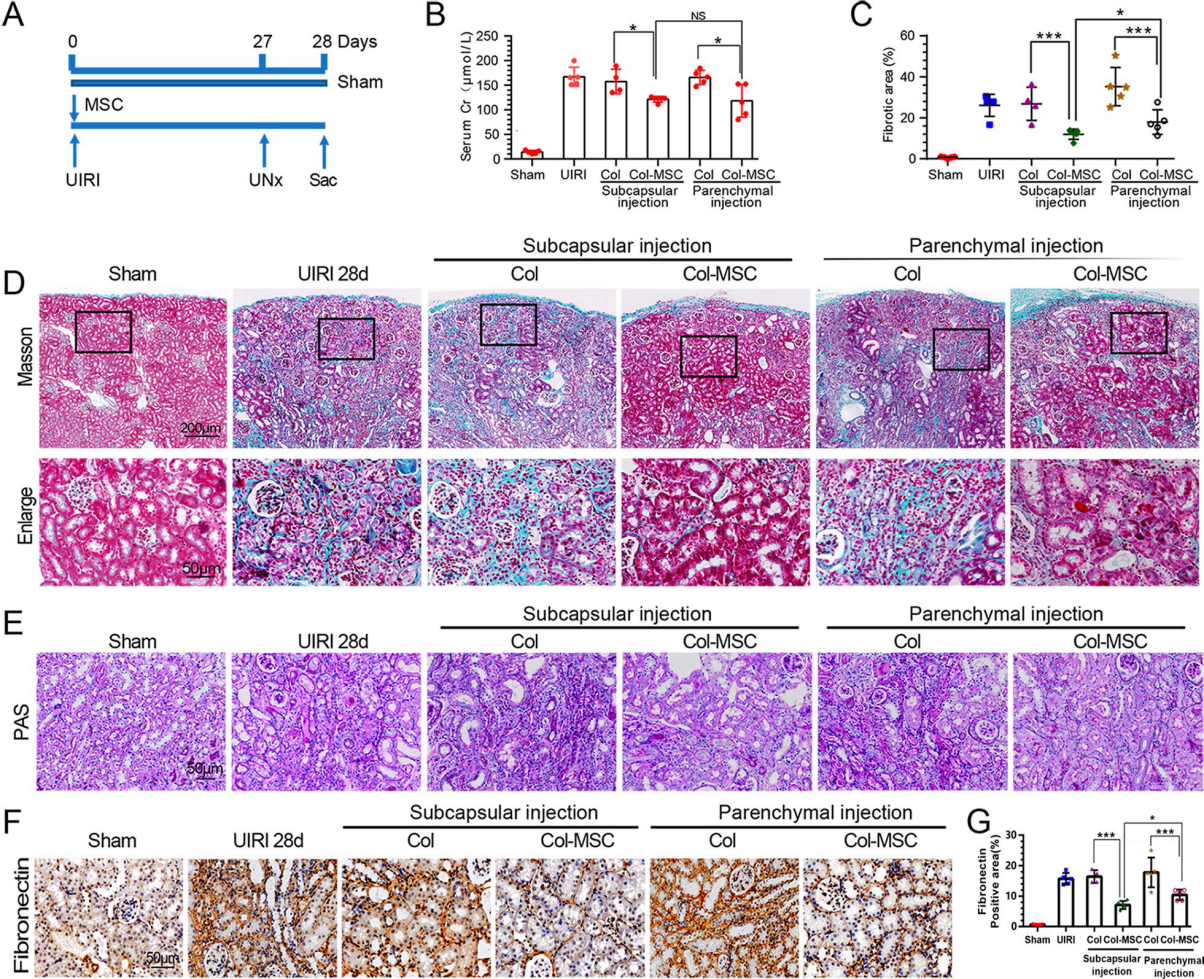
Differences in the antifibrotic effects of subcapsular and parenchymal MSC transplantation in AKI-CKD mice at 28 days. **A** Experimental design. **B** Serum creatinine levels in the groups at 28 days after IRI. ***p < 0.001, ANOVA corrected with the Bonferroni coefficient. *n* = 4–5 mice per group. **C** Quantitative assessment of fibrotic areas. **p < 0.01; *p < 0.05, ANOVA corrected with the Bonferroni coefficient. *n* = 4–5 mice per group. **D** Representative micrographs showing Masson staining of renal collagen deposition at 28 days after IRI in the various groups as indicated. **E** Representative micrographs showing PAS staining in the various groups. **F** Representative immunohistochemical staining of fibronectin in the groups after 28 days of IRI. **G** Quantitative analysis of the fibronectin-positive areas in the groups after IRI. ***p < 0.001, ANOVA corrected with the Bonferroni coefficient. *n* = 4–5 per group

To understand the influence of subcapsular and parenchymal MSC transplantation on the AKI-to-CKD transition, we examined the long-term responses of mouse kidneys following IRI (Fig. 5A and Additional file 1: Supplementary Figure 2A). A significant decrease in SCr was observed after Col-MSC injection relative to that observed upon Col injection at 14 and 28 days after IRI. However, there was no significant difference in SCr levels between the subcapsular and parenchymal Col-MSC transplantation groups (Fig. 5B and Additional file 1: Supplementary Figure 2B). At 14 days, focal atrophic tubules began to appear in the IRI and IRI + Col groups as the kidneys transitioned to CKD (Additional file 1: Supplementary Figure 2E). At 28 days, CKD developed with areas of tubular atrophy, interstitial fibrosis, and chronic inflammation in the abovementioned groups (Fig. 5E). MSCs transplanted by both local delivery routes alleviated tubular atrophy and chronic inflammation at 14 and 28 days, with better positive effects in the subcapsular MSC transplantation groups than the other groups (Fig. 5E and Additional file 1: Supplementary Figure 2E).

ECM accumulation (collagen deposition) was initially assessed by Masson’s trichrome staining. Compared with those in the IRI and IRI + Col groups, fewer fibrotic areas were observed in both Col-MSC-transplanted groups at 14 and 28 days. The subcapsular Col-MSC group had a lower fibrotic area percentage than the parenchymal group on Day 28 (Fig. 5C, D). Fibronectin and α-SMA staining were carried out to confirm the antifibrotic effects of MSCs. MSC transplantation reduced fibronectin- and α-SMA-positive areas at 14 and 28 days (Fig. 5F, G and Additional file 1: Supplementary Figure 2F-H). The percentages of the fibronectin-positive area in the subcapsular MSC-transplanted groups were lower than those of the parenchymal MSC-transplanted groups at 28 days.

## Discussion

**Fig. 6:**
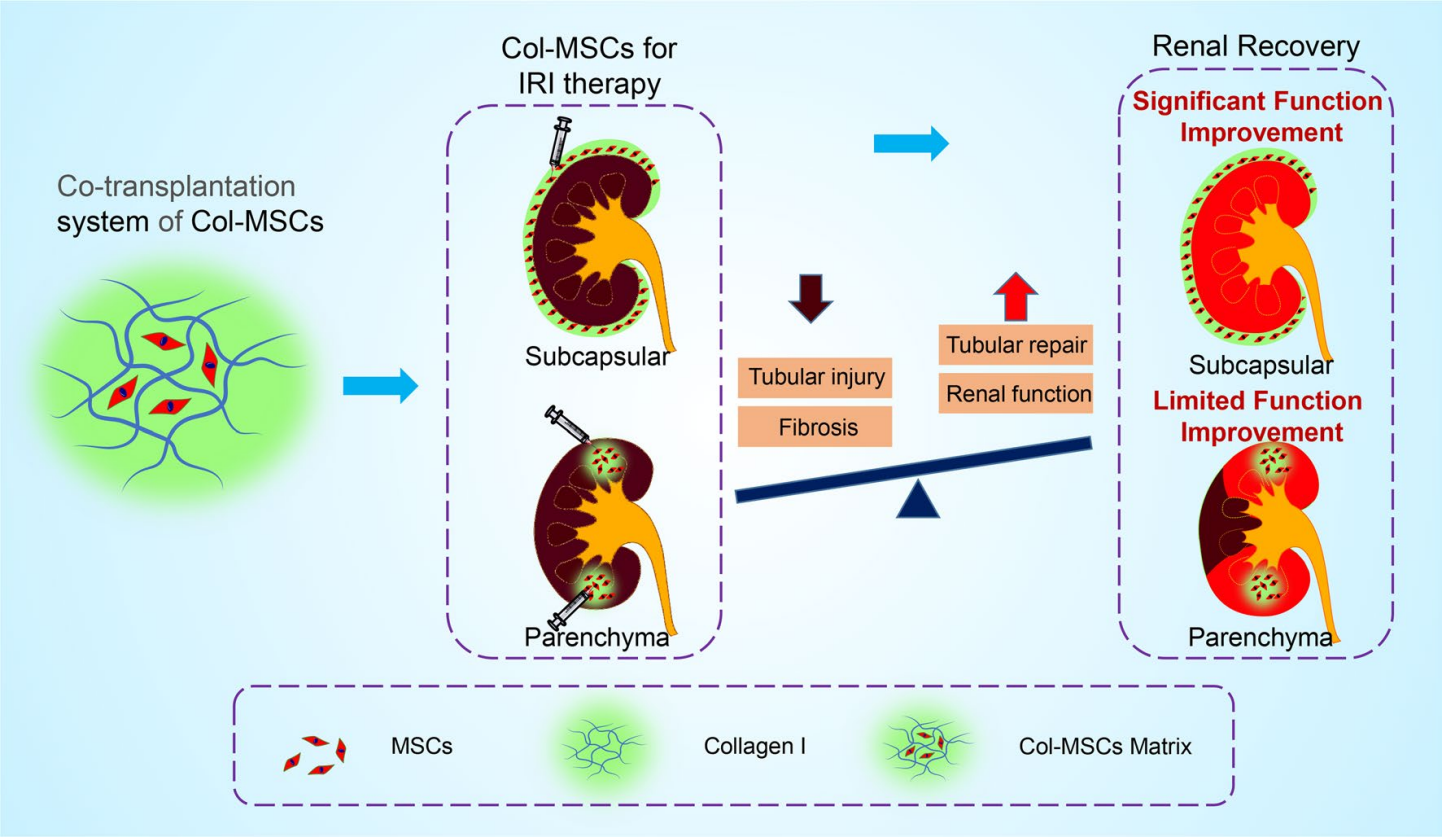
Schematic illustration depicting the treatment efficacies of Col-MSC transplantation via the renal subcapsular and parenchymal routes in AKI-CKD mice

Although numerous studies have shown beneficial effects of MSC-based therapy for AKI-CKD, there are controversies regarding the administration strategies. Therefore, we investigated the effects of cell transplantation routes on a mouse model of AKI-to-CKD induced by ischemia/reperfusion. To the best of our knowledge, this is the first study to compare the treatment efficacy of MSCs administered locally to the subrenal capsule or parenchyma in a preclinical AKI model (Fig. 6).

In the present study, the therapeutic effects of the subcapsular and parenchymal routes of MSC transplantation were compared from 1 to 28 days using the same dose and cell suspension volume. Both local therapies were able to effectively reduce tubular injury and promote tubular epithelial cell repair and regeneration after AKI on Day 3 and alleviate renal fibrosis beginning on Day 14, suppressing AKI-to-CKD progression. In addition, we found that MSC administration via the subcapsular route may be more efficient for renal structural and functional recovery than MSC administration via the parenchymal route. There may be two main reasons for this difference in efficacy. First, kidney damage caused by AKI is usually diffuse. Single-point injection of MSCs into the renal parenchyma is limited to certain injury sites, resulting in local kidney repair, which has a limited effect on promoting the recovery of overall renal function and structure. However, the subcapsular cavity is composed of the space between the renal parenchyma and the fibrous membrane that envelops the entire kidney, and MSCs injected under the capsule can cover almost the entire surface of the renal parenchyma, resulting in therapeutic effects in the entire kidney. We used black ink to mimic the diffusion range of the MSC suspension under the renal capsule and into the parenchyma. After the same amount of ink was injected (as shown in Additional file 1: Supplementary Figure 3), the ink diffused under the capsule, almost covering the entire kidney surface, whereas the diffusion range of ink injected into the parenchyma was limited, which confirmed the previous point. Second, there are abundant blood vessels under the renal capsule, and the growth factors and chemokines secreted by the transplanted MSCs can be quickly absorbed by the surrounding blood vessels to promote renal repair and inhibit fibrosis.

Interestingly, the rapid protective effect of MSCs injected by both local routes was not observed within the first 24 h of AKI in our study. There were no differences in renal function or ATN scores among the IRI model group, IRI + Col I groups, and IRI + Col-MSC groups at 24 h (Additional file 1: Supplementary Figure 4). This may be because MSCs need to be digested, centrifuged, washed, and mixed with collagen matrix at low temperatures before transplantation. After transplantation in the kidney, it takes almost 24 h for MSCs to return to a normal state before they can exert positive effects (Fig. 1B and Additional file 2: Supplementary Video 1).

The kidney is a dense tissue, and the space in the renal parenchyma or under the renal capsule is relatively limited. If a stem cell suspension is directly injected into the kidney in a liquid state, it will easily leak out due to the high internal pressure, and very few stem cells will remain in the kidney (Additional file 3: Supplementary Video 2). In addition, due to the lack of cell–matrix interactions after transplantation, the poor microenvironment also leads to rapid apoptosis, which ultimately fails to achieve the desired therapeutic effect. Collagen matrix is a typical three-dimensional crosslinking reticulated porous structure with high adhesion that has been applied in tissue repair postinjury as an ideal delivery system for cells and drugs. Collagen matrix is heat sensitive, a liquid at 4 °C and a solid at 37 °C. Once encapsulated in the collagen matrix, MSCs can be captured by the crosslinking network (Fig. 1A) and can grow and proliferate well to exert better therapeutic effects in the natural ECM microenvironment that is mimicked by collagen I in vitro. In this study, we mixed MSCs with the collagen matrix and injected the suspension into the renal capsule and parenchyma. The solid-state can help reduce the leakage of MSCs and collagen matrix stabilize the retention rate of MSCs in the kidney for at least 14 days. In addition, we also compared damaged kidney structure and function between the IRI + Col I group (collagen I injection alone) and the model IRI group at the same time and found that except for renal parenchymal damage at the injection site due to the needle, there was no difference in kidney damage between the collagen group and the IRI group. This finding suggested that collagen itself does not damage the kidney and is a promising candidate tissue support material for AKI treatment.

Although renal parenchymal or subcapsular route can trap MSCs in the injured region and have little adverse effect on the whole body, local transplantation also has drawbacks. Renal parenchymal injection can damage the kidney tissue at the injection site (Additional file 1: Supplementary Figure 5), while subrenal capsule injection is difficult to perform due to the thin capsule, and it is often necessary to expose the kidney by open operation.

Further researches are encouraged to overcome the limitation of local transplantation. With the continuous progress in imaging medicine, we may try to use imaging technology to guide subcapsular and parenchymal delivery in the future to minimize kidney damage [31].

## Conclusion

The present study demonstrates that MSC administration via the subcapsular route may be more efficient for functional recovery after AKI than MSC administration via the parenchymal route, and collagen I can provide a better microenvironment for cell–cell and cell–matrix interactions to stabilize the retention rate of MSCs in the kidney.

## Supplementary Information

**Additional file 1: Supplementary Figure 1.** Surgical transplantation of Col-MSCs under the renal capsule or into the parenchyma in a mouse model of IRI.**Supplementary Figure 2**. Differences in the antifibrotic effects of subcapsular and parenchymal MSC transplantation in AKI-CKD mice at 14 days.**Supplementary Figure 3**. Gross images of ink diffusion after injection via the subcapsular and parenchymal routes.**Supplementary Figure 4**. Changes in kidney pathological damage after MSC transplantation via the subcapsular and parenchymal routes at 24h. **Supplementary Figure 5**. The injured kidney tissue at the injection site by renal parenchymal route.

**Additional file 2.** The growth and proliferation of RFP-MSCs in collagen matrix were observed continuously and dynamically for 84 hours by high-content screening.

**Additional file 3.** The video of MSCs injected into the renal capsule in a liquid state.MSCs easily leaked out due to the high internal pressure, and very few stem cells remained in the kidney.

## Data Availability

All data generated and/or analyzed during this study are available from the corresponding author upon reasonable request.

## Abbreviations

MSCs: Mesenchymal stem cells
AKI: Acute kidney injury
CKD: Chronic kidney disease
ECM: Extracellular matrix
Col-MSCs: Collagen matrix-encapsulated-MSCs
IRI: Ischemia/reperfusion injury
RFP-MSCs: Red fluorescent protein-labeled MSCs
ATN: Acute tubular necrosis
SCr: Serum creatinine
PAS: Periodic acid-Schiff
