# Supplementary material for: Comparison of the treatment efficacy of umbilical mesenchymal stem cell transplantation via renal subcapsular and parenchymal routes in AKI-CKD mice
Source: Stem Cell Res Ther. 2022 Mar 25;13:128. doi: 10.1186/s13287-022-02805-3 (PMC8953025; doi:10.1186/s13287-022-02805-3)
Supplement: Supplementary file 1 — Additional file 1: Supplementary Figure 1. Surgical transplantation of Col-MSCs under the renal capsule or into the parenchyma in a mouse model of IRI.Supplementary Figure 2. Differences in the antifibrotic effects of subcapsular and parenchymal MSC transplantation in AKI-CKD mice at 14 days.Supplementary Figure 3. Gross images of ink diffusion after injection via the subcapsular and parenchymal routes.Supplementary Figure 4. Changes in kidney pathological damage after MSC transplantation via the subcapsular and parenchymal routes at 24h. Supplementary Figure 5. The injured kidney tissue at the injection site by renal parenchymal route. [file 13287_2022_2805_MOESM1_ESM.docx]

**Additional file 1**

Supplementary Figure 1: Surgical transplantation of Col-MSCs under the renal capsule or into the parenchyma in a mouse model of IRI.

Supplementary Figure 2: Differences in the antifibrotic effects of subcapsular and parenchymal MSC transplantation in AKI-CKD mice at 14 days.

Supplementary Figure 3: Gross images of ink diffusion after injection via the subcapsular and parenchymal routes.

Supplementary Figure 4: Changes in kidney pathological damage after MSC transplantation via the subcapsular and parenchymal routes at 24h.

Supplementary Figure 5: The injured kidney tissue at the injection site by renal parenchymal route.


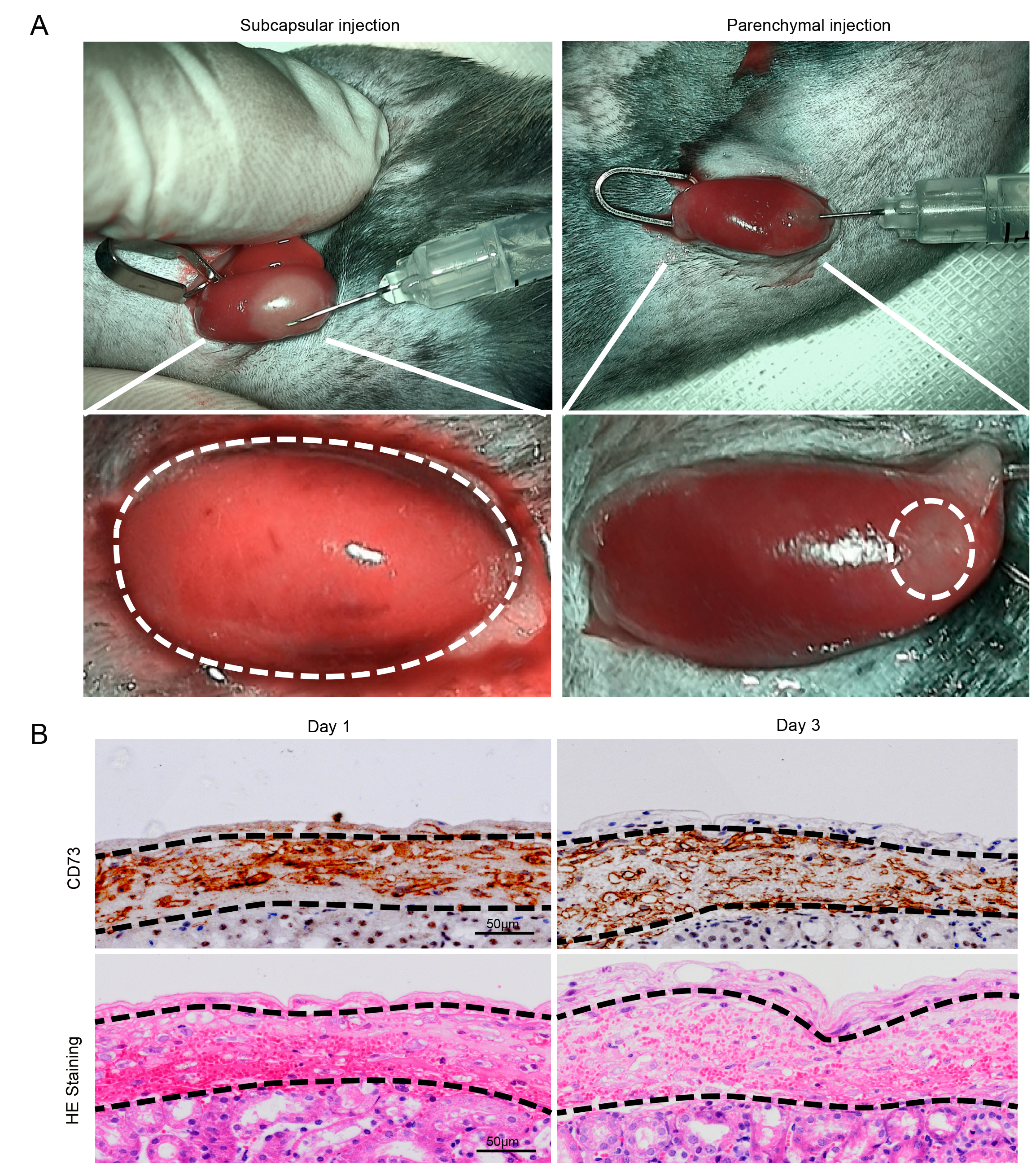


**Supplementary Figure 1.** **Surgical** **transplantation of** **Col-MSCs** **under the** **renal capsule or into the** **parenchyma in a mouse model of IRI.**

(A) Illustration showing surgical subcapsular and parenchymal MSC transplantation. The white dotted line shows the range of MSC distribution.

(B) Immunohistochemical staining of CD73-positive cells (CD73: a marker of HUC-MSCs) and HE staining were observed under the renal capsule on Day 1 and 3. The blue dotted lines indicated the edges of the MSCs under the renal capsule. Scale bar = 50 μm.


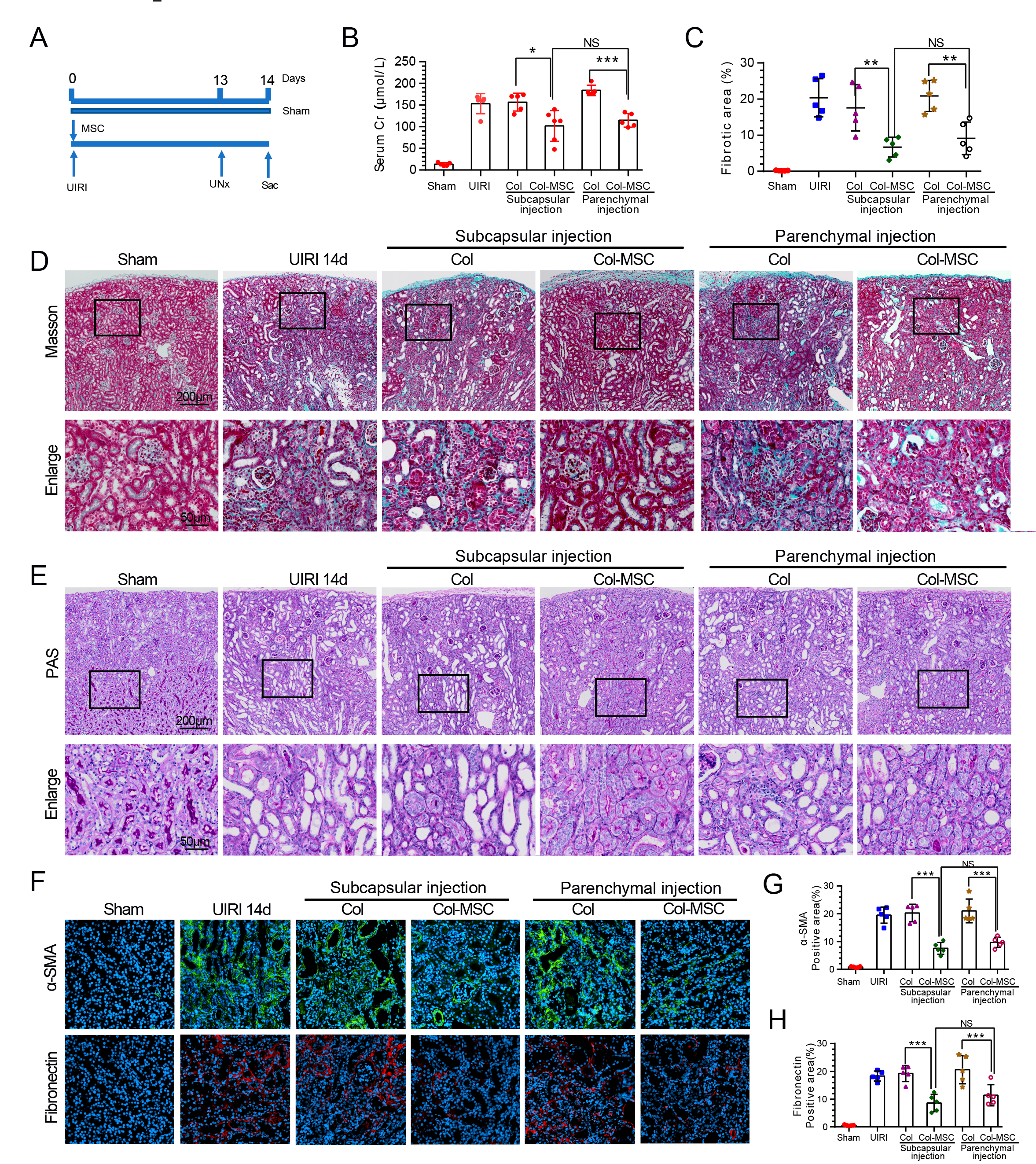


**Supplementary Figure 2. Differences in the antifibrotic effects of subcapsular and parenchymal MSC transplantation in AKI-CKD mice at 14 days.**

(A) Experimental design.

(B) Serum creatinine levels in the groups at 14 days after IRI. ***p< 0.001, ANOVA corrected with the Bonferroni coefficient. n = 5-6 mice per group.

(C) Quantitative assessment of fibrotic areas. **p< 0.01, *p< 0.05, ANOVA corrected with the Bonferroni coefficient. n = 5 mice per group.

(D) Representative micrographs following Masson staining of renal collagen deposition at 14 days after IRI in the groups as indicated.

(E) Representative micrographs showing PAS staining in the various groups.

(F) Representative immunofluorescence staining of α-SMA (green) and fibronectin (red) in the groups after 14 days of IRI.

(G) Quantitative analysis of the α-SMA- and fibronectin-positive areas in the groups after IRI. ***p< 0.001, ANOVA corrected with the Bonferroni coefficient. n=5 per group.


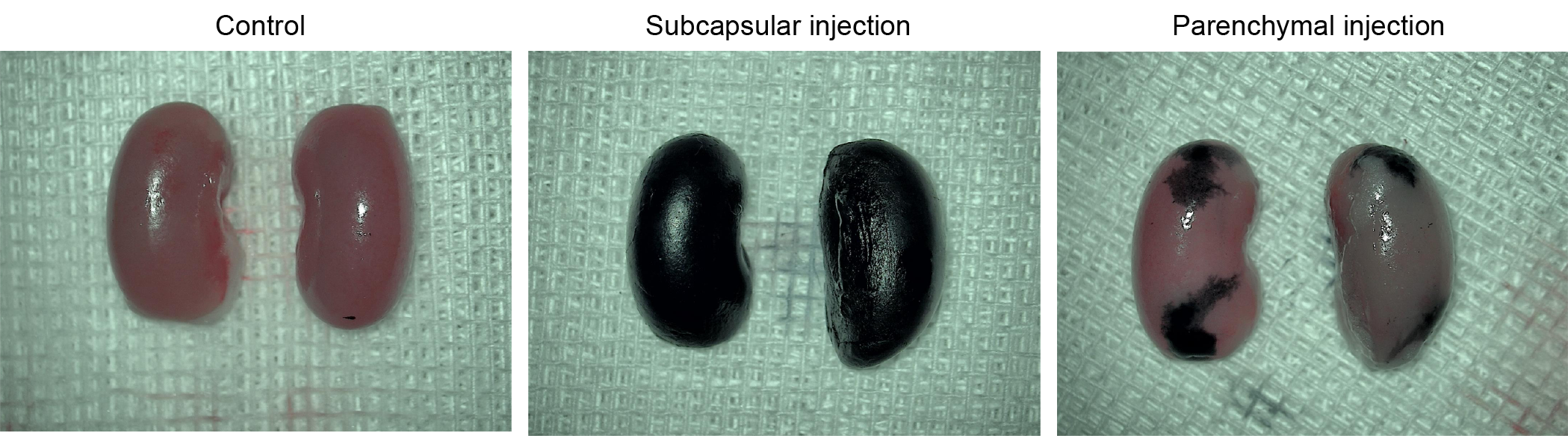


**Supplementary Figure 3.** **Gross images of ink diffusion after injection via the subcapsular and parenchymal routes.**

In isolated kidneys, ink was delivered to the subrenal capsule and parenchyma to mimic the distribution of MSCs.


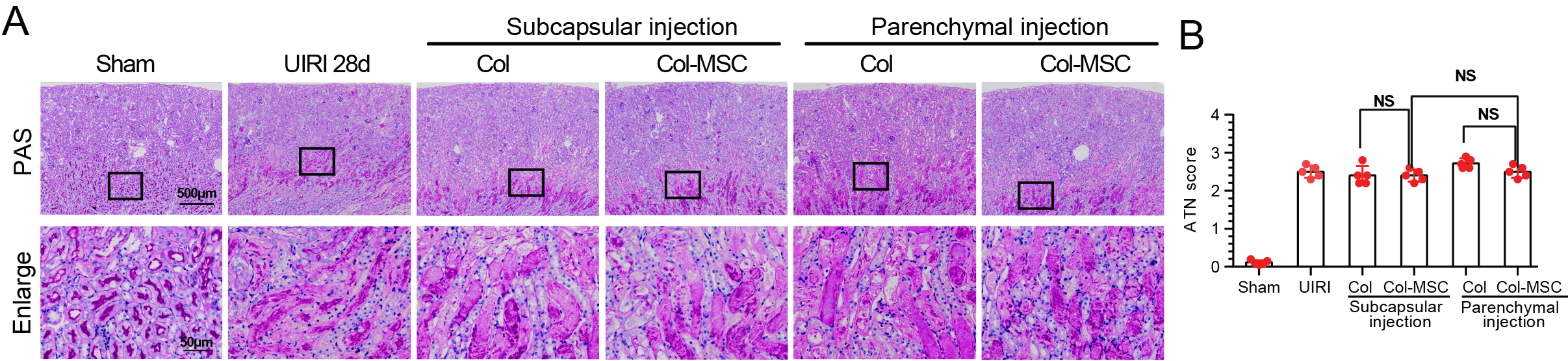


**Supplementary Figure 4. Changes in kidney pathological damage** **after MSC transplantation via the subcapsular and parenchymal routes at 24h.**

(A) Representative micrographs of PAS staining showing kidney injury in IRI mice after subcapsular and parenchymal MSC transplantation.

(B) Quantitative assessment of tubular damage. n=5 mice per group.


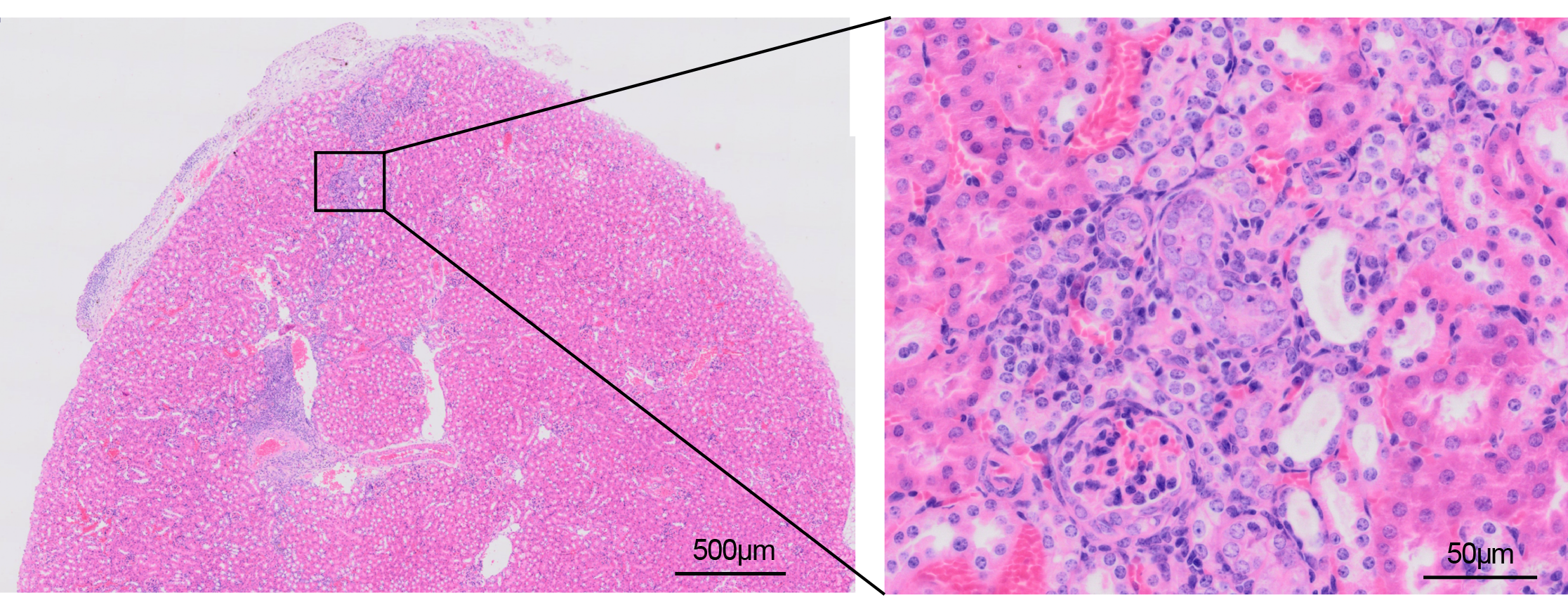


**Supplementary Figure 5. The injured kidney tissue at the injection site by renal parenchymal route.**
